# Levels of CEACAM6 in Peripheral Blood Are Elevated in Patients with Plasma Cell Disorders: A Potential New Diagnostic Marker and a New Therapeutic Target?

**DOI:** 10.1155/2019/1806034

**Published:** 2019-01-27

**Authors:** N. Steiner, R. Hajek, D. Nachbaur, B. Borjan, S. Sevcikova, G. Göbel, E. Gunsilius

**Affiliations:** ^1^Laboratory for Tumor Biology & Angiogenesis, Department of Internal Medicine V (Hematology and Medical Oncology), Medical University of Innsbruck, Innsbruck, Austria; ^2^Faculty of Medicine, University of Ostrava, Ostrava, Czech Republic; ^3^Department of Hematooncology, University Hospital Ostrava, Ostrava, Czech Republic; ^4^Department of Internal Medicine V (Hematology and Medical Oncology), Medical University of Innsbruck, Innsbruck, Austria; ^5^Babak Myeloma Group, Department of Pathological Physiology, Faculty of Medicine, Masaryk University, Brno, Czech Republic; ^6^Department of Clinical Hematology, University Hospital Brno, Brno, Czech Republic; ^7^Department of Medical Statistics, Informatics and Health Economics, Medical University of Innsbruck, Innsbruck, Austria

## Abstract

**Introduction:**

The prognosis of multiple myeloma is still unfavorable due to inherent characteristics of the disease and the often-delayed diagnosis due to widespread and unspecific symptoms such as back pain and fatigue. Therefore, a simple diagnostic blood test would be helpful to speed up the diagnostic procedure in such patients (pts.). Here, we evaluated the diagnostic value of plasma levels of carcinoembryonic antigen-related cell adhesion molecule 6 (CEACAM6) in the peripheral blood and bone marrow of pts. with plasma cell disorders and in healthy controls.

**Materials and Methods:**

Immunoreactive CEACAM6 was determined in the peripheral blood and bone marrow (*n* = 95/100) of pts. with monoclonal gammopathy of unknown significance (MGUS: 28/37), newly diagnosed multiple myeloma (NDMM: 42/40), and relapsed/refractory multiple myeloma (RRMM: 25/23) by sandwich ELISA.

**Results:**

Median CEACAM6 levels in the peripheral blood of pts. with plasma cell disorders were significantly higher than those of healthy controls (healthy controls: 15.2 pg/ml (12.1-17.1); MGUS: 19.0 pg/ml (16.4-22.5); NDMM: 18.0 pg/ml (13.4-21.2); and RRMM: 18.9 pg/ml (15.2-21.5); *p* < 0.001). Plasma levels of CEACAM6 discriminated healthy subjects from MGUS/NDMM pts. (AUC = 0.71, 95% CI: 0.6-0.8); i.e., a CEACAM6 level > 17.3 pg/ml has an 82% (95% CI: 70-90) predictive probability for the identification of MGUS or NDMM. Moreover, CEACAM6 levels in the bone marrow were significantly higher in RRMM pts. than in NDMM pts. (*p* = 0.04), suggesting a role of this molecule in disease progression.

**Conclusion:**

CEACAM6 plasma levels can noninvasively identify pts. with a plasma cell disorder and should be evaluated prospectively as a potential diagnostic marker. Moreover, due to high CEACAM6 levels in the bone marrow in RRMM pts., this adhesion molecule might be a therapeutic target in multiple myeloma pts.

## 1. Introduction

Multiple myeloma (MM) is a highly heterogeneous, incurable plasma cell malignancy. The often-delayed diagnosis due to widespread and unspecific symptoms such as back pain and fatigue causes substantial morbidity, so that new accurate biomarkers in the blood to speed up the diagnostic procedure in such pts. are needed.

Carcinoembryonic antigen-related cell adhesion molecule 6 (CEACAM6), a member of the immunoglobulin superfamily, is overexpressed in various cancers, such as breast, non-small-cell lung, ovarian, and colon cancer [1–3]. This transmembrane glycoprotein can mediate cell signaling pathways involved in tumor development and progression, angiogenesis, antiapoptosis, and drug resistance. Zang et al. showed a role of CEACAM6 in promotion of tumor angiogenesis and vasculogenic mimicry in gastric cancer via FAK signaling [4]. In gastric cancer, CEACAM6 was proposed as a prognostic biomarker and potential therapeutic target [5]. Levels of CEACAM6 DNA detected by RT-PCR in peripheral blood cells of gastric cancer pts. correlated with disease stage, suggesting CEACAM6 as a marker for early diagnosis of metastasis and recurrence [2]. Duxbury et al. found increased CAECAM6 expression as a parameter for an adverse clinical outcome in pancreatic adenocarcinoma [6]. Analysis of peripheral blood revealed elevated CEACAM6 serum protein levels in pancreatic ductal adenocarcinoma (PDAC) pts. compared to chronic pancreatitis and healthy blood donors. Increased CEACAM6 values in serum of PDAC pts. were associated with the presence of distant metastasis and tumor grading [7]. Moreover, an anti-CEACAM6 antibody inhibited pancreatic cancer cells [8]. In contrast to elevated CEACAM6 tissue expression, mRNA levels in peripheral blood of colorectal cancer appeared to be lower than those in normal healthy blood [9]. CEACAM6-targeting antibodies are in preclinical development to treat various solid cancers [10, 11].

Multiple myeloma cells expressing CEACAM6 can inhibit myeloma-specific CD8^+^ T cell reactivity and cytotoxicity [12].

Here, we evaluated the diagnostic value of plasma levels of CEACAM6 in the peripheral blood and bone marrow of pts. with plasma cell disorders and in healthy controls.

## 2. Materials and Methods

### 2.1. Ethics Statement

The research has been conducted in accordance with the Declaration of Helsinki and has been approved by the ethics committee of the Medical University of Innsbruck and Brno (Innsbruck: number AN2015-0034 346/4.13 and number 5064; Brno: 20/1/2011).

### 2.2. Patients and Sample Collection

Peripheral blood samples of healthy controls (*n* = 41) pts. with MGUS (*n* = 28 peripheral blood (PB) samples/37 bone marrow (BM) plasma samples), NDMM (*n* = 42 PB/40 BM), and RRMM (*n* = 25 PB/23 BM) were analyzed. Diagnostic criteria were according to the International Myeloma Working Group (IMWG) [13]. Patient characteristics are shown in Table 1. Peripheral and bone marrow blood samples underwent centrifugation for 10 min at 1000 ×*g*, and the obtained plasma was collected and stored at -80°C.

### 2.3. Sandwich ELISA for CEACAM6

We followed the methods of Steiner et al. [14]. A commercially available human CEACAM6 ELISA kit, purchased from LifeSpan BioSciences, was used according to the manufacturer's instructions. In the assay based on sandwich ELISA principle, no significant cross-reactivity or interference between targeted protein CEACAM6 and CEACAM2, CEACAM4, or CEACAM7 was observed. Capture and detection antibodies, both polyclonal rabbits, were purified by antigen-specific chromatography followed by protein A. The titer (affinity) of capture antibody in ELISA was 3.6 × 10^5^ and of detection antibody was 2 × 10^5^. Plasma samples, diluted 3-fold with the assay buffer, were incubated in duplicates in the ELISA plate precoated with specific capture antibody for 2.5 hours at room temperature [14]. Four washes were performed to remove unbound proteins. Each well was incubated with a biotin-labeled specific detection antibody at room temperature for 1 hour [14]. After the washing steps for removal of unbound antibody, the plate was treated with a streptavidin-horseradish peroxidase conjugate for 45 min [14]. Following an enzymatic reaction with the substrate for peroxidase (room temperature, 30 min) and subsequent termination of the color development, the absorbance at 450 nm and 550 nm was measured using an automated microplate reader [14].

### 2.4. Statistical Analysis

Descriptive data are shown using frequencies, median, and interquartile range. We used mainly nonparametric tests (Wilcoxon test, Kruskal-Wallis test) to identify differences of CEACAM6 levels between the groups, and we provide original *p* values without corrections for multiple testing. Spearman correlation coefficient was used to test associations between quantitative variables. Sensitivity, specificity, and thresholds were calculated by ROC analysis and area under the curves (AUCs) using Youden's approach. All tests for statistical significance were two-sided. Confidence intervals and *p* values were calculated at a 5% level of significance. Statistical evaluation was performed using SPSS statistical software (version 24.0; SPSS Inc., Chicago, IL, USA) and OpenEpi v3.

## 3. Results

### 3.1. CEACAM6 Levels in Peripheral Blood Are Higher in Plasma Cell Disorders than in Healthy Controls

**Figure 1:**
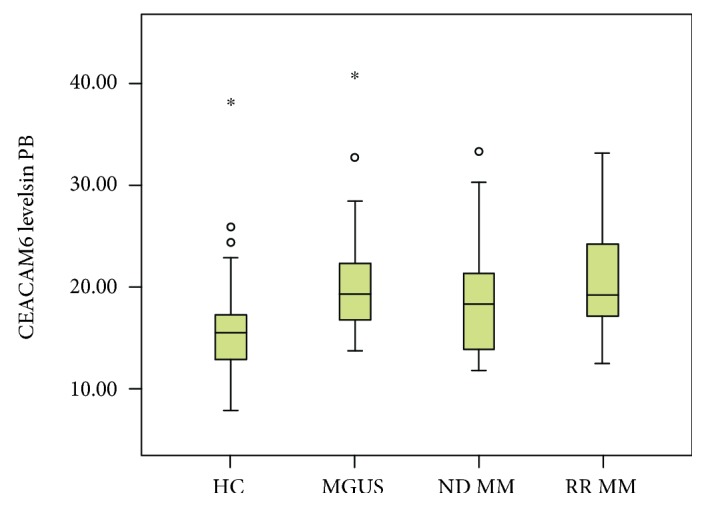
CEACAM6 levels in peripheral blood (PB) of healthy controls (HC): MGUS, RRMM, and NDMM pts. *p* values below a significance level of *α* = 0.05 were considered as statistically significant.

A marked difference of CEACAM6 in peripheral blood was observed between healthy controls (median 15.2 pg/ml, IQR 12.1-17.1), MGUS pts. (19.0/16.4-22.5), NDMM pts. (18.0/13.4-21.2), and RRMM pts. (18.9/16.5-24.1) (*p* < 0.001, Figure 1). Post hoc tests revealed that median CEACAM6 levels in the control group were significantly lower than those in all the patient groups (Table 2).

### 3.2. High CEACAM6 Levels in Peripheral Blood Identify Patients with Plasma Cell Disorders

**Figure 2:**
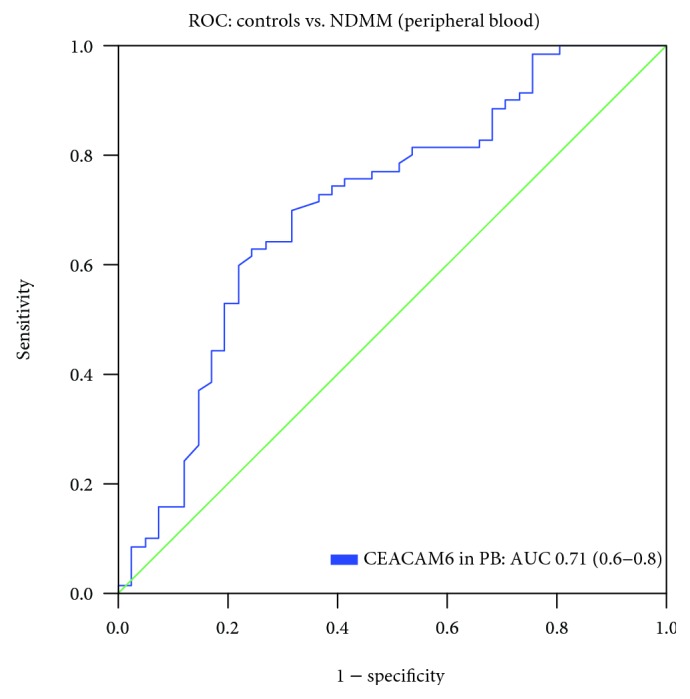
Receiver operating curve (ROC) analyses for peripheral blood (PB) plasma CEACAM6 to differentiate healthy controls vs. NDMM pts.

We calculated the receiver operating curve (ROC) by plotting sensitivity against specificity within controls and plasma cell disorders and used the overall median as threshold. Peripheral blood plasma levels of CEACAM6 discriminate healthy subjects from MGUS/NDMM pts. (AUC = 0.71, 95% CI: 0.6-0.8) (Figure 2). A peripheral blood plasma CEACAM6 value greater than the median value of 17.3 pg/ml was suitable to identify pts. with plasma cell dyscrasias. Individuals with elevated CEACAM6 levels (>17.3 pg/ml) faced an 82% (95% CI: 70-90) predictive probability to have MGUS or NDMM. The negative predictive value (npV) for being healthy in this cohort was 53% (95% CI: 41-65). The sensitivity was 60% (95% CI: 48-71) and the specificity was 78% (95% CI: 63-88).

### 3.3. Bone Marrow CEACAM6 Levels Are Higher in RRMM than in NDMM Patients

**Figure 3:**
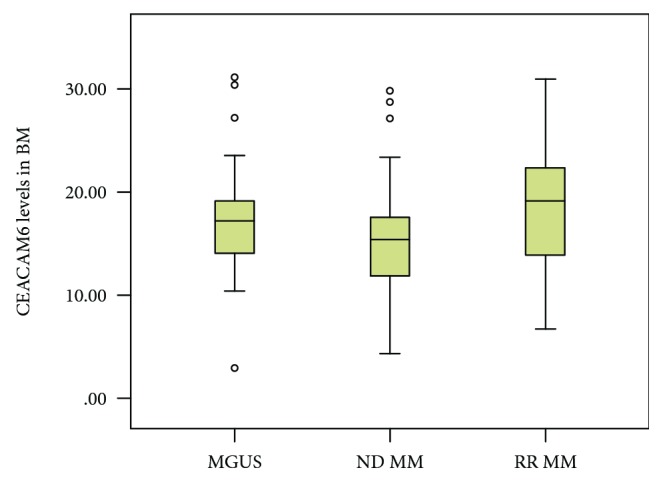
CEACAM6 levels in the bone marrow (BM) between MGUS, RRMM, and NDMM pts. *p* values below a significance level of *α* = 0.05 were considered as statistically significant.

**Figure 4:**
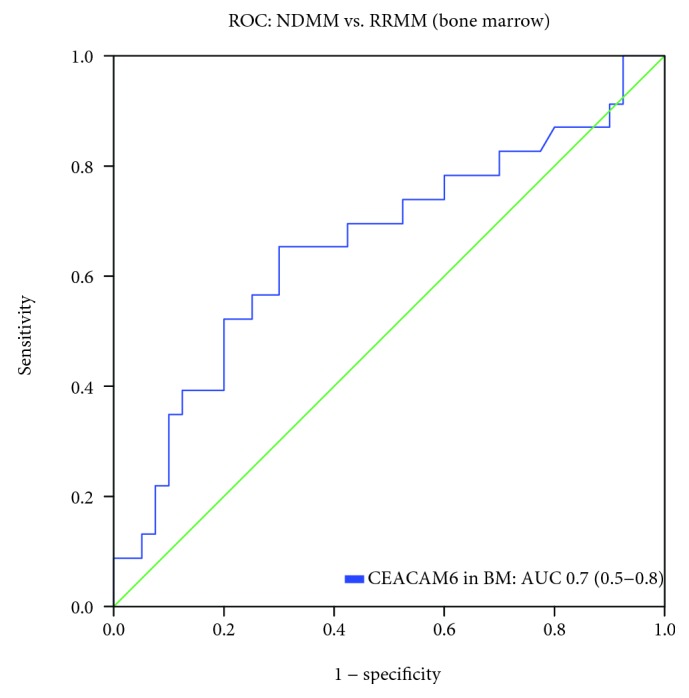
Receiver operating curve (ROC) analyses for bone marrow (BM) plasma CEACAM6 to differentiate NDMM vs. RRMM pts.

Bone marrow plasma CEACAM6 levels were higher in RRMM pts. than in pts. with NDMM (median 18.9 pg/ml, IQR 14.0-22.9; *p* = 0.04). The comparison between MGUS, NDMM, and RRMM was not significantly different (*p* = 0.07, Figure 3 and Table 3). The discriminative AUC between the NDMM and RRMM groups yielded 0.66 (95% CI: 0.51-0.81) (Figure 4). The peak sensitivity based on a threshold of 16.7 pg/ml was 57% (CI 37-74), and the specificity was 70% (CI 55-82). This threshold showed a negative predictive value of 74% (CI 58-85), whereas the positive predictivity was low at 52% (CI 34-70).

## 4. Discussion

Delays in the diagnosis of multiple myeloma are not uncommon due to the nonspecific symptomatology in these patients resembling widespread diseases as back pain, fatigue, and susceptibility to infections. A delay in the diagnosis of MM is associated with an unfavorable clinical course [15]. Graziani et al. described latencies in establishing the diagnosis of ≥12 months in multiple myeloma pts. [16]. Of note, in up to 35% of the pts., the median time from first symptoms to the diagnosis of MM was ≥12 months in a prospective study [16].

Therefore, a new accurate biomarker, easily measurable in peripheral blood, would be helpful to speed up the diagnostic procedure, which thereby can reduce morbidity and mortality in these pts. Here, we analyzed CEACAM6 in the peripheral and bone marrow blood of healthy controls and pts. with plasma cell disorders by sandwich ELISA and found that CEACAM6 plasma levels can noninvasively identify pts. with a plasma cell disorder. Moreover, the high levels of CEACAM6 in the bone marrow of RRMM pts. suggest a role of this molecule in the progression of MM.

CEACAM6 (carcinoembryonic antigen-related cell adhesion molecule 6) belongs to the carcinoembryonic antigen (CEA) family of glycosylphosphatidylinositol- (GPI-) anchored cell surface glycoproteins and plays a role in tumor cell migration, invasion, adhesion, and formation of distant metastases [17]. Solid tumors, such as colorectal, ovarian, non-small-cell lung, and breast cancer, show elevated CAECAM6 expression [1–3]. Increased expression of CEACAM6 promotes tumor metastasis, antiapoptosis, and angiogenesis in pts. with advanced-stage gastric cancer [18]. Overexpression of CAECAM6 is associated with an aggressive phenotype mediated via TGF*β*, AKT, FAK, and SRC signaling [19]. In vitro studies have shown the therapeutic potential of an antibody directed against CEACAM6 in lung adenocarcinoma as an adjuvant therapy combined with paclitaxel [10]. Preliminary results show an activity of CEACAM6-targeting antibodies in human malignancies [8, 12, 19, 20]. Moreover, CEACAM6 can decrease the host immune response when overexpressed in myeloma cells [12].

## 5. Conclusion

We show here that CEACAM6 levels of >17.3 pg/ml identify pts. with a plasma cell disease with an 82% predictive probability for MGUS or NDMM. Thus, the quantification of CEACAM6 levels in the blood might be a valuable diagnostic tool to speed up the diagnosis of a malignant plasma cell disorder in individuals with widespread, uncharacteristic clinical symptoms and thus potentially decreasing morbidity and even mortality. A prospective study referring to this is underway.

Moreover, the higher levels in the bone marrow of RRMM pts. compared to NDMM pts. suggest that CEACAM6 is involved in myeloma disease progression, though the mechanisms are still subject to investigation.

## Figures and Tables

**Table 1:** Patient demographics and characteristics (*n* = 103).

Parameter	MGUS		NDMM		RRMM	
	*n* = 38	%	*n* = 42	%	*n* = 25	%
Median age (range) (years)	66 (58-74)		73 (61-79)		62 (53-68)	
Sex						
F	14	31	19	42	12	27
M	24	40	23	38	13	22
ISS stage						
I	n.a.		8	50	8	50
II	n.a.		11	58	8	42
III	n.a.		23	72	9	28
Type of Ig heavy chain						
IgG	20	35	21	36	17	29
IgM	13	87	0	0	2	13
IgA	3	25	8	67	1	8
IgD	0	0	1	100	0	0
Light chain only	2	11	12	63	5	26
Type of Ig light chain						
Kappa	23	35	27	42	15	23
Lambda	15	37.5	15	37.5	10	25
*β*-2 microglobulin>UNV	17	25	33	48	19	27
LDH>UNV	5	26	6	32	8	42
Creatinine ≥ 1.3 mg/dl	12	26	25	53	10	21
Serum calcium>UNV	1	8	8	61	4	31
Haemoglobin ≤ 12 g/dl	15	22	34	50	19	28
Osteolytic bone lesions	1	2	35	57	25	41
Cytogenetic standard risk	4	19	13	62	4	19
Cytogenetic high risk	3	8	20	50	17	42
Cytogenetic not available	31	70	9	21	4	9
Plasma cells in BM (median; range)	3 (1.5-5)		30 (7.2-70)		50 (18-80)	
Therapy lines at sample collection						
1-2	1	11	2	22	6	67
≥3	0	0	0	0	19	100
PI-based therapy	0	0	0	0	11	100
IMiD-based therapy	1	9	2	18	8	73
PI+IMiD-based therapy	0	0	0	0	3	100
Other therapies	0	0	0	0	3	100
No therapy	37	48	40	52	0	0

*n*: number of patients; ISS: international staging system; Ig: immunoglobulin; UNV: upper normal value; LDH: lactate dehydrogenase; IMiD: immunomodulatory drugs; PI: proteasome inhibitor; BM: bone marrow; n.a.: not applicable. Cytogenetic defined high-risk disease (i.e., del17p13, t(4;14), t(14;16), t(14/20), and 1q21 gain). All others are considered standard risk.

**Table 2:** CEACAM6 levels in peripheral blood are increasing from healthy controls to MGUS/MM pts.

Patients	CEACAM6 levels	CEACAM6 levels	CEACAM6 levels	*p* value
	Median (pg/ml)	95% CI	IQR	
Healthy con. (*n* = 41)	15.2	13.8–16.3	12.1–17.1	
MGUS (*n* = 28)	19.0	16.9–20.6	16.4–22.5	
NDMM (*n* = 42)	18.0	16.2–19.8	13.4–21.2	
RRMM (*n* = 25)	18.9	16.9–22.2	16.5–24.1	
All pts. (*n* = 136)	17.3	16.4–18.4	16.4–18.4	*p* < 0.001

Healthy con.: healthy controls; MGUS: monoclonal gammopathy of undetermined significance; MM: multiple myeloma; NDMM: newly diagnosed multiple myeloma; RRMM: relapsed/refractory multiple myeloma; IQR: interquartile range.

**Table 3:** CEACAM6 levels in the bone marrow of MGUS, NDMM, and RRMM pts.

Patients	CEACAM6 levels	CEACAM6 levels	CEACAM6 levels	*p* value
	Median (pg/ml)	95% CI	IQR	
MGUS (*n* = 37)	17.5	14.9–18.7	14.1–19.4	
NDMM (*n* = 40)	14.9	13.3–16.5	12.2–18.2	
RRMM (*n* = 23)	18.9	15.3–22.1	14.0–22.9	
All pts. (*n* = 100)	16.2	14.9–17.5	13.3–19.7	*p* = 0.07

MGUS: monoclonal gammopathy of undetermined significance; MM: multiple myeloma; NDMM: newly diagnosed multiple myeloma; RRMM: relapsed/refractory multiple myeloma; IQR: interquartile range.

## Data Availability

The data used to support the findings of this study are included within the article.

## References

[1] Jantscheff P., Terracciano L., Lowy A. (2003). Expression of CEACAM6 in resectable colorectal cancer: a factor of independent prognostic significance. *Journal of Clinical Oncology*.

[2] Zhao Z. S., Li L., Wang H. J., Wang Y. Y. (2011). Expression and prognostic significance of CEACAM6, ITGB1, and CYR61 in peripheral blood of patients with gastric cancer. *Journal of Surgical Oncology*.

[3] Blumenthal R. D., Leon E., Hansen H. J., Goldenberg D. M. (2007). Expression patterns of CEACAM5 and CEACAM6 in primary and metastatic cancers. *BMC Cancer*.

[4] Zang M., Zhang Y., Zhang B. (2015). CEACAM6 promotes tumor angiogenesis and vasculogenic mimicry in gastric cancer *via* FAK signaling. *Biochimica et Biophysica Acta (BBA) - Molecular Basis of Disease*.

[5] Ru G. Q., Han Y., Wang W. (2017). CEACAM6 is a prognostic biomarker and potential therapeutic target for gastric carcinoma. *Oncotarget*.

[6] Duxbury M. S., Matros E., Clancy T. (2005). CEACAM6 is a novel biomarker in pancreatic adenocarcinoma and PanIN lesions. *Annals of Surgery*.

[7] Gebauer F., Wicklein D., Horst J. (2014). Carcinoembryonic antigen-related cell adhesion molecules (CEACAM) 1, 5 and 6 as biomarkers in pancreatic cancer. *PLoS One*.

[8] Cheng T. M., Murad Y. M., Chang C. C. (2014). Single domain antibody against carcinoembryonic antigen-related cell adhesion molecule 6 (CEACAM6) inhibits proliferation, migration, invasion and angiogenesis of pancreatic cancer cells. *European Journal of Cancer*.

[9] Rodia M. T., Ugolini G., Mattei G. (2016). Systematic large-scale meta-analysis identifies a panel of two mRNAs as blood biomarkers for colorectal cancer detection. *Oncotarget*.

[10] Hong K. P., Shin M. H., Yoon S. (2015). Therapeutic effect of anti CEACAM6 monoclonal antibody against lung adenocarcinoma by enhancing anoikis sensitivity. *Biomaterials*.

[11] Niu G., Murad Y. M., Gao H. (2012). Molecular targeting of CEACAM6 using antibody probes of different sizes. *Journal of Controlled Release*.

[12] Witzens-Harig M., Hose D., Junger S. (2013). Tumor cells in multiple myeloma patients inhibit myeloma-reactive T cells through carcinoembryonic antigen-related cell adhesion molecule-6. *Blood*.

[13] The International Myeloma Working Group (2003). Criteria for the classification of monoclonal gammopathies, multiple myeloma and related disorders: a report of the International Myeloma Working Group. *British Journal of Haematology*.

[14] Steiner N., Hajek R., Sevcikova S. (2017). High levels of FLT3-ligand in bone marrow and peripheral blood of patients with advanced multiple myeloma. *PLoS One*.

[15] Kariyawasan C. C., Hughes D. A., Jayatillake M. M., Mehta A. B. (2007). Multiple myeloma: causes and consequences of delay in diagnosis. *QJM: An International Journal of Medicine*.

[16] Graziani G., Herget G. W., Ihorst G. (2017). Time from first symptom onset to the final diagnosis of multiple myeloma — possible risks and future solutions: large retrospective and confirmatory prospective ‘Deutsche Studiengruppe Multiples Myelom’ (DSMM) analysis. *Blood*.

[17] Blumenthal R. D., Hansen H. J., Goldenberg D. M. (2005). Inhibition of adhesion, invasion, and metastasis by antibodies targeting CEACAM6 (NCA-90) and CEACAM5 (carcinoembryonic antigen). *Cancer Research*.

[18] Zang M., Hu L., Cao S. (2017). Dual role of carcinoembryonic antigen-related cell adhesion molecule 6 expression in predicting the overall survival of gastric cancer patients. *Scientific Reports*.

[19] Johnson B., Mahadevan D. (2015). Emerging role and targeting of carcinoembryonic antigen-related cell adhesion molecule 6 (CEACAM6) in human malignancies. *Clinical Cancer Drugs*.

[20] Imakiire T., Kuroki M., Shibaguchi H. (2004). Generation, immunologic characterization and antitumor effects of human monoclonal antibodies for carcinoembryonic antigen. *International Journal of Cancer*.

